# Changing food choices: the option for high-protein foods and the move away from the Mediterranean diet

**DOI:** 10.1007/s40519-024-01668-2

**Published:** 2024-06-04

**Authors:** Concetta M. Vaccaro, Giulia Guarino, Francesco Danza, Alessia Fraulino, Renata Bracale

**Affiliations:** 1 Censis Foundation, Health and Welfare Department, Piazza di Novella, 2, Rome, Italy; 2 Department of Medicine and Health Sciences, University of Molise, Campobasso, Italy; 3 Circana s.r.l., Viale Cassala, 22, Milan, Italy

**Keywords:** Protein foods, Mediterranean diet, Health, Fitness, Cardiovascular risk

## Abstract

**Purpose:**

The growing importance placed on health and physical well-being by consumers continues to influence food industry choices. The food market therefore, pandering to the desires for a lean and athletic body, offers new products deemed more healthy and able to impact body image. It is evidenced, thus, a change in food choices and habits, with more attention to the quality and nutrient content of the products consumed, in which protein is assuming increasing importance. The purpose of the study is to highlight important changes in eating habits and in particular the increase in the consumption of high-protein foods, attributable to the focus on physical fitness and thinness, resulting in a decreasing adherence to the Mediterranean diet and the progressive loss of its positive impact on health.

**Methods and results:**

This analysis is based on CIRCANA srl data on food consumption trends (change percentage of quantity and value sales) in recent years. Specifically, between January and September 2022 vs. 2021, there was a 21.6% increase in the sale of high-protein products, significantly higher than all the previous ones.

**Conclusions:**

The past few years have seen the gradual discovery of new products, at first little-known and niche, which are becoming major players on the national food consumption scene. The trend is toward a growing preference for high-protein foods and diets with the gradual abandonment of the Mediterranean and an increased risk of nutritional deficiencies, obesity, and cardiovascular disease.

**Level of evidence:**

Level IV, evidence obtained from multiple time series with or without the intervention.

## Introduction

The sometimes excessive focus on wellness and fitness has led to a change in food choices and habits, altering the profile of the Mediterranean Diet characteristic of our country.

The background cultural fact is the growing awareness that lifestyles are a key determinant of good health: this is a belief now shared by 75.8% of Italians, according to findings in a 2022 Censis survey, up from 41.1% found in 2016 [1]. It is a conviction that also translates into concrete behaviors, with significant shares of Italians claiming to follow healthy lifestyles based on a commitment to good nutrition, some form of physical activity and control of risk factors such as smoking and alcohol (82.1% of Italians claim to do so in at least one area). Moreover, this is a choice that appears to be more widespread among the younger generations, with the figure ranging from 88.4% of those aged 18–34 vs. 78.5% relating to the over64s [2]. In the various beliefs around what constitutes a healthy diet, about which inaccuracies and confusion are frequently found [3], very often those who shape their diets, in order to lose weight or improve sports performance, first and foremost seek protein with prominent interest [4].

Protein is an essential nutritional component of the diet, as it ensures growth, supports muscle and bone metabolism, ensures the maintenance and development of a normal nervous system, and helps sustain muscle mass and physical performance [4].

The Reference Intake Levels of Nutrients and Energy (LARN), compiled by the Italian Society of Human Nutrition, indicate 0.9 g per kilogram of body weight as the daily amount of protein needed by our body [5]. Unlike low-active consumers, athletes may have high physiological protein requirements, as the intensity and duration of athletic performance increases, to maintain adequate protein synthesis and energy production [4].

In 2016, the Academy of Nutrition and Dietetics (AND), Dietitians of Canada (DC) and the American College of Sports Medicine (ACSM) published their joint position on proper nutrition to support athletic performance. They stated that the level of protein intake for athletes should be between 1.2 and 2.0 g/kg/day [6]. The increase in protein recommendations for athletes, coupled with the idea that high protein intake improves muscle mass, muscle function and body weight, has led the general population to believe that consuming foods that are naturally rich and/or enriched in protein is “healthy” and ensures fitness [4].

These needs have, therefore, driven the growth of the food industry’s supply of new products defined as “high protein,” but often not in line with the definition of high-protein products given in EC Regulation 1924/2006.^1^

Protein diet is not synonymous with healthy diet, yet, the axiom is a common belief among consumers, which helps explain the increasing attention and consumption of foods defined as protein and high-protein [4]. Habits thus deviate from the traditional Mediterranean diet characterized by a high intake of plant-based foods such as fruits, vegetables, legumes, whole grains, nuts and seeds, as well as moderate consumption of fish, seafood, dairy products, poultry and eggs. Frequent use of extra virgin olive oil as a dietary source of fat and moderate consumption of wine with meals are among its mainstays, while consumption of red and processed meats is limited [7].

## Methods

This analysis is based on CIRCANA srl data on food consumption trends in Italy (percentage increase in sales in value and quantity) between January and September 2022 compared with 2021, and with trends in previous years. The information refers to all channels of modern organized distribution (Hypermarkets, Supermarkets, Free Service, Traditional and Discount). The data collected refer to the new “High Protein” trend and to foods that are naturally source of protein, the “Classic Proteins.” In particular, to select foods with high protein content, the company CIRCANA Ltd. chose to start from the definitions provided by the companies and highlighted in the “marketing claims” that intend to indicate a high protein content of the product. From an “I imagine” database provided by I imagine-Gs1, the following were chosen as claims: High Protein, Protein Burger, HiPRO, MILKPRO, Protein Rich. In contrast, keywords such as: Source of Protein, 16 g of Protein per packet, Right amount of protein.

## Results

Data on quantity and value sales trends of protein products at the indicated distribution channels highlight the significant growth of both Classic Protein and High Protein products between 2019 and 2021.

**Fig. 1:**
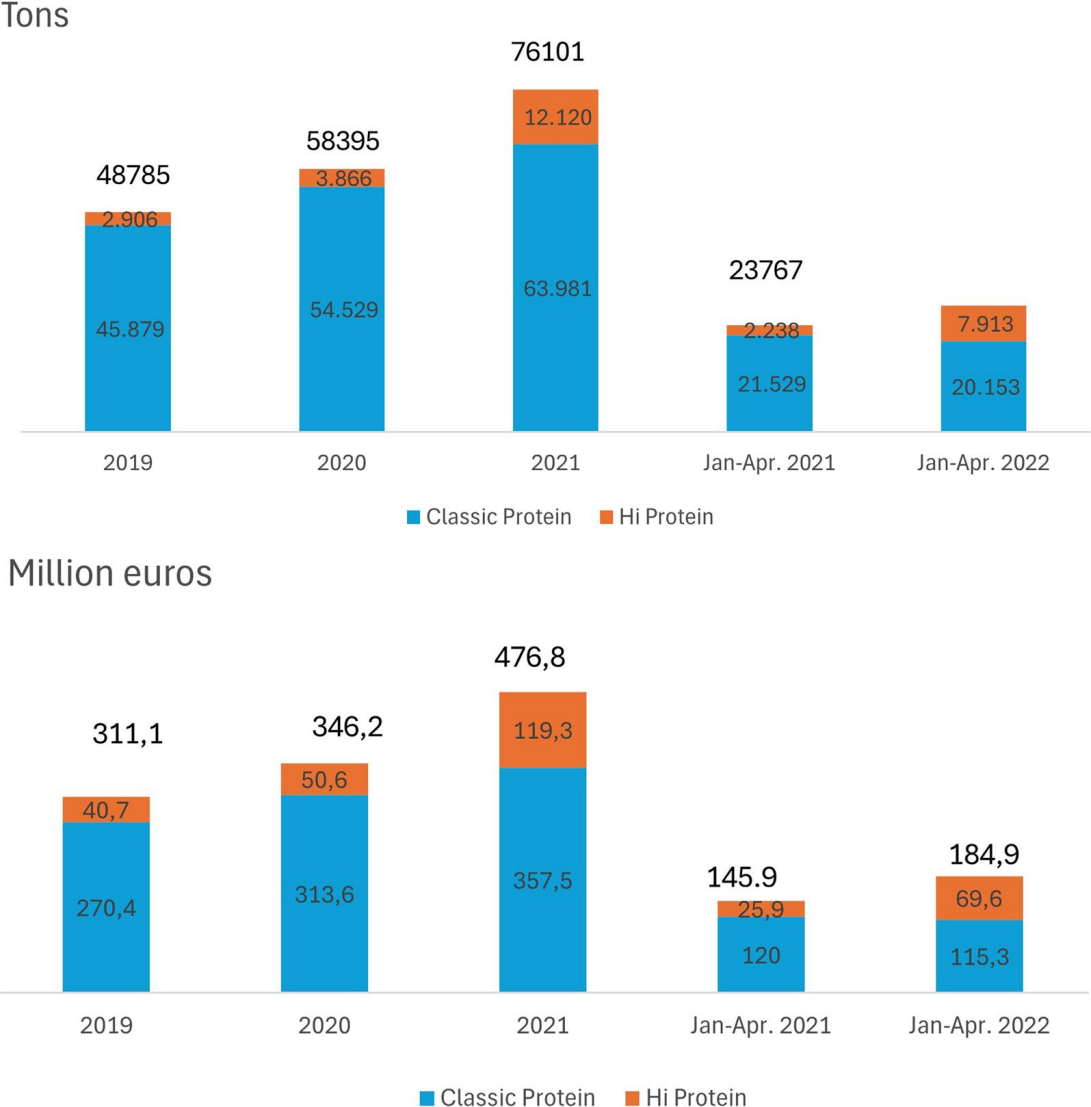
Trend of “Classic Protein” and “High Protein” products (in volume and value) 2019–April 2022 (Source: Circana srl)

In addition, considering the most recent data, updated to April 2022, it can be seen that against an overall increase in volume sales of the protein market of 18.1% between January and April 2022 compared to the same period in 2021, it is the High protein segment that has driven the growth (+ 253.6% vs. − 6.4 in Classic protein). Similar is the trend in value (+ 26.8% overall, -3.9% in Classic protein and + 169.0% in High protein) (Fig. 1).

**Fig. 2:**
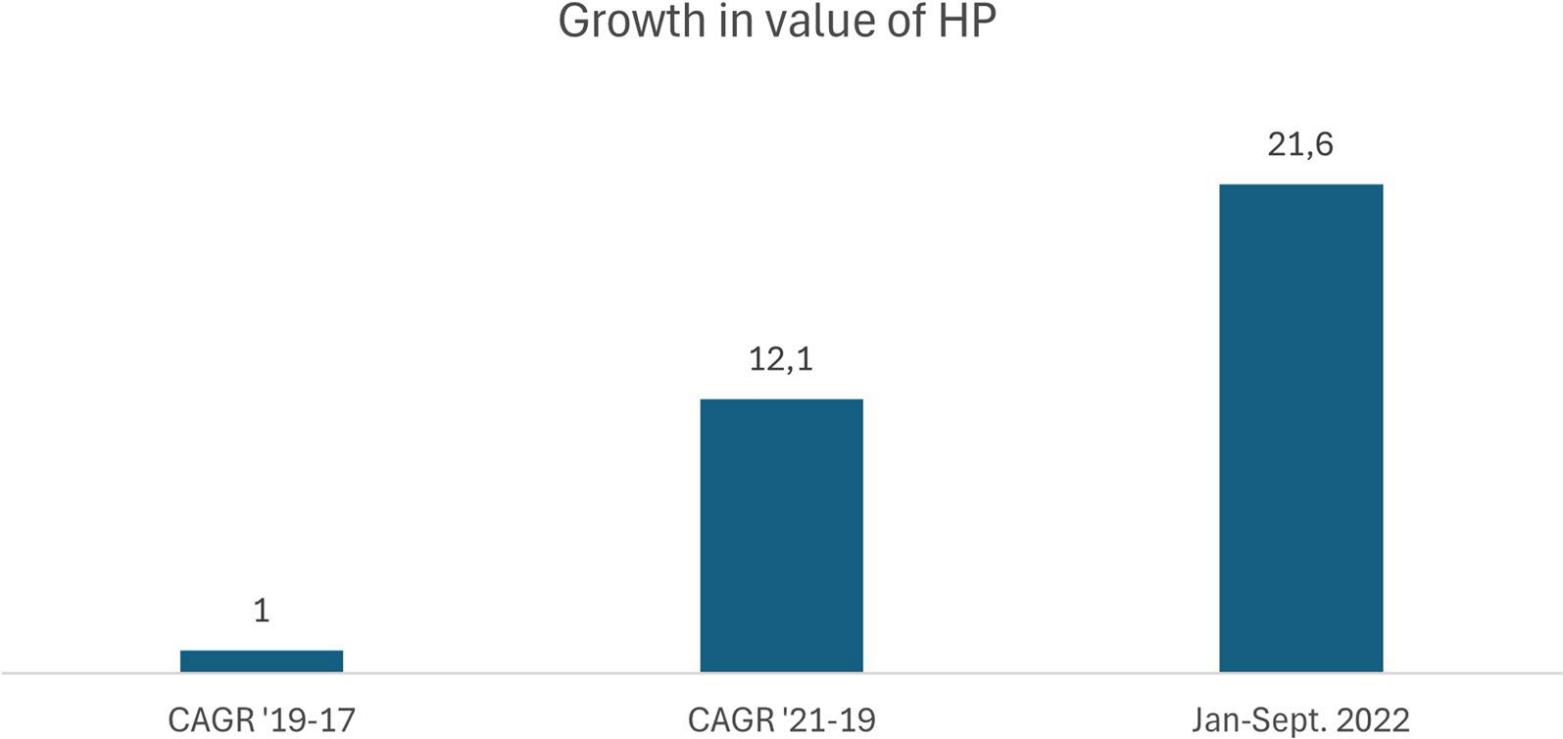
Average percentage growth (CAGR Compounded Average Growth Rate) of value sales of “High Protein” products 2017–September 2022 (Source: Circana srl)

Moreover, analysis of the trend in value (average percentage growth) of High protein products alone, extended through September 2022, highlights not only the significance of the increase, but also the novelty of this market expansion: the value increase recorded in the time intervals considered went from + 1.0% in 2019–2017 to + 12.1% in 2021–2019 to 21.6% in January–September 2022, highlighting a transformation of the food landscape, with the rapid spread of these new products, including yogurt, bars, cereals, supplements, cheeses, spreads, but also beverages (Fig. 2).

In addition, considering the 15 “Hight Protein” categories by revenue growth, the first place is occupied by fresh protein desserts, which recorded a revenue growth of 48.4% between 2021 and 2022 (Jan.–Nov.2022). These are mostly products launched in the Oct.–Nov.2021 period, which have captured growing slices of the market in a relatively short time. Finally, the High protein strand brings with it interest in products, naturally a source of protein, hitherto present but in low demand in the Italian market, as signaled by the increase in value sales of peanut butter products, which showed a 22.8% increase in value sales in the period between May and September 2022 alone, compared to an average growth of 16.2% in the 3-year period 2021–2019.

## Discussion

The pursuit of health and well-being has involved increasing shares of the population in recent years, leading to an increasing attention to dietary habits, performance and physical fitness [2]. In this new scenario, there has recently been a marked increase in the importance attached to protein intake, involving a wide range of people, from professional athletes to overweight and obese individuals [8].

The belief in the health and fitness benefits associated with the consumption of such products is precisely reflected in the data on food market trends, which show a rapid and significant increase in sales especially of innovative high protein products.

These new trends are accompanied by a gradual abandonment of the healthy principles of the Mediterranean diet, as also reported by Godos [9], who reported a significant decline in the last decade in adherence to it by the general population. A shift away from Mediterranean habits has been detected even in the younger segment of the population as highlighted by Mattavelli et al. [10]. Data from this research, from different Italian regions [obtained using the Mediterranean diet scale (MDS)] and related to a sample of 565 adolescents aged 12–19 years, highlight low adherence to the Mediterranean diet in 38.6% of the subjects, while only 14% show high adherence [10]. Since exhaustive evidence demonstrates the central and preventive role of the Mediterranean diet, it is clear that a departure from it may have long-term health effects, increasing cardiovascular risk, obesity, metabolic syndrome and type 2 diabetes, as well as of certain types of neurodegenerative diseases, cognitive disorders in old age, and cancers [3, 7].

The continued decline in adherence rates to the Mediterranean diet represents a global threat not only in terms of population health status but also in terms of environmental sustainability. The environmental advantage that characterizes the Mediterranean diet, linked to the ability to enhance typical local products, made by small/medium-sized enterprises, is being countered by an economically competitive and increasingly globalized food market that does not always guarantees the same quality standards in terms of product [7]

### Strengths and limitations

The strength of the present study lies in the presentation of unpublished and objective data on the consumption of food and in particular of high protein products. The analysis conducted in the course of this research allowed us to fully capture the new emerging trend, identifying its distinctive characteristics and the mutually reinforcing effect between consumer beliefs and food choices and commercial offerings. On the other hand, the limitation lies in the absence of data referring to age and more generally to the socio-anagraphic characteristics of consumers. Further research is needed to obtain a complete and in-depth view. Moreover, the scientific literature currently seems to lack specific data linking the consumption of high-protein products to negative health effects. While this is a limitation of our work, it is also an incentive to further investigate this fundamental aspect.

## Conclusions

The positive cultural trend that values healthy lifestyles and individual commitment to pursuing them suffers from many informational uncertainties, which are accompanied by food consumption choices that may be counterproductive to their intended health goal. One example is the increasing consumption of high-protein products, which may be inappropriate for those without higher protein requirements, such as athletes. Moreover, these new consumption options are often accompanied by lower adherence to the Mediterranean diet.

Taking scientific evidence into account, future research and action should support better public awareness of the health and environmental benefits associated with increased adherence to the Mediterranean diet, including highlighting the risks associated with poor information about proper nutrition and the resulting market choices. Correct information and the involvement of professionals in the field is indeed essential to follow a healthy and balanced diet that can at the same time meet any increased needs and guarantee the nutritional benefits of a proper diet.

### What is already known about this topic?

It is now well-known that interest in appearance and fitness has increased significantly over the years. The goal of achieving a lean, athletic body has influenced people’s eating choices and habits.

### What does this study add?

Our study presented an innovative perspective based on analysis of objective data on the characteristics of Italians’ food consumption, which highlighted a recent and significant trend of inclusion in Italians’ diets of products defined as high-protein, often at the expense of the dictates of the Mediterranean diet, with a counterproductive effect precisely with respect to the health goal intended there.

## Data Availability

There is no data access link for this paper. Data will be made on request.

## Footnotes

1 According to Regulation (EC) 1924/2006, a food can be defined as: 1) "source of protein" if its protein component provides at least 12% of the calories. total; 2) "high in protein" if the contribution to total calories of said component reaches. at least 20%.
